# Pharmacokinetic parameters of ifosfamide in mouse pre-administered with grapefruit juice or naringin

**DOI:** 10.1038/s41598-019-53204-3

**Published:** 2019-11-12

**Authors:** Eduardo Madrigal-Bujaidar, Edilberto Pérez-Montoya, Sandra García-Medina, José Melesio Cristóbal-Luna, José A. Morales-González, Eduardo Osiris Madrigal-Santillán, Rogelio Paniagua-Pérez, Isela Álvarez-González

**Affiliations:** 1 Laboratorio de Genética, Escuela Nacional de Ciencias Biológicas, Instituto Politécnico Nacional. Av. Wilfrido Massieu s/n, Col. Zacatenco, Del. Gustavo A. Madero, Ciudad de México, 07738 Mexico; 2 Laboratorio de Biofarmacia, Departamento de Farmacia, Escuela Nacional de Ciencias Biológicas, Instituto Politécnico Nacional, Av. Wilfrido Massieu s/n, Col. Zacatenco, Del. Gustavo A. Madero, Ciudad de México, 07738 Mexico; 3 Laboratorio de Medicina de la Conservación, Escuela Superior de Medicina, Instituto Politécnico Nacional. Plan de San Luis y Díaz Mirón s/n, Col. Casco de Santo Tomás, Del. Miguel Hidalgo, Ciudad de México, 11340 Mexico; 4 Instituto Nacional de Rehabilitación, Servicio de Bioquímica. Av. México-Xochimilco 289, Ciudad de México, 14389 Mexico

**Keywords:** Analytical biochemistry, Biological techniques

## Abstract

Grapefruit juice (GFJ) and naringin when consumed previously or together with medications may alter their bioavailavility and consequently the clinical effect. Ifosfamide (IF) is an antitumoral agent prescribed against various types of cancer. Nevertheless, there is no information regarding its interaction with the ingestion of GFJ or naringin. The aims of the present report were validating a method for the quantitation of IF in the plasma of mouse, and determine if mice pretreated with GFJ or naringin may modify the IF pharmacokinetics. Our HPLC results to quantify IF showed adequate intra and inter-day precision (RSD < 15%) and accuracy (RE < 15%) indicating reliability. Also, the administration of GFJ or naringin increased *C*_*max*_ of IF 22.9% and 17.8%, respectively, and decreased *T*_*max*_ of IF 19.2 and 53.8%, respectively. The concentration of IF was higher when GFJ (71.35 ± 3.5 µg/mL) was administered with respect to that obtained in the combination naringin with IF (64.12 ± µg/mL); however, the time required to reach such concentration was significantly lower when naringin was administered (p < 0.5). We concluded that pre-administering GFJ and naringin to mice increased the *T*_*max*_ and decreased the *C*_*max*_ of IF.

## Introduction

Grapefruit juice (GFJ) is a complex mixture formed by a number of compounds that include minerals, vitamins, flavonoids, furanocoumarins and sesquiterpenes, among other chemicals. The juice is known to have nutritional importance as well as to possess a number of biomedical properties, including a strong antioxidant activity, and effects related with the decrease of metabolic syndrome symptoms, digestive problems, and with the improvement of cardiac diseases. Moreover, it has also been reported its effect in the prevention of chronic degenerative diseases, and cancer^1–4^. One of the constituents of GFJ, the flavanone naringin has been identified as the responsible for the bitter flavor of the fruit. The chemical however, has also been found in other citrus fruits and teas, as well as in plants such as cocoa, beans, oregano or tomato^5–7^. Naringin has also been reported to have pharmacologic effects on similar disease conditions to those mentioned for GFJ, besides antibiotic and anti-parasitic properties^8,9^.

Interestingly, both agents (GFJ and naringin) have shown the capacity to modify the pharmacokinetic parameters of numerous medications when ingested together with, or close to the consumption of the juice or of the flavanone. Bailey *et al*.^10^ were the first group that described such drug interaction, and informed on a significant increase in the bioavailability of felodipine and nifedipine. In the following years, a number of interactions between drugs and GFJ or naringin consumption has been detected presenting alterations of pharmacokinetic parameters. Such interactions have been observed with the use of calcium channel blockers, HMG-CoA reductase inhibitors, immunosuppressant agents, inhibitors of HIV proteases, phosphodiesterase type 5 inhibitors, antihistamine, anthelmintic, and anti-inflammatory drugs^10–19^. The intervention of GFJ and naringin in such processes has been found in various *in vitro* and *in vivo* assays, as well as in computational modeling studies. These studies have suggested at least two mechanisms involved to explain the observed effects: the inhibition of intestinal enzymes (mainly CYP3A) to decrease the rate of pre-systemic metabolism, and consequently to increase the bioavailability of the involved drug^20,21^, and alterations in molecular transporters, such as the organic anion-transporting polypeptide 2B1 (OATP2B1), the multidrug-resistant protein sulfotransferases 1 and 3, and the P-glycoprotein transporter^22,23^. An interesting report by Dresser *et al*.^24^, demonstrated that GFJ is a potent inhibitor of OATP, therefore may modify the absorption of drugs and consequently their bioavailability. Besides, a decrease in drug uptake transport has been generally related with the inhibition of organic anion transporting polypeptides, as well as with the inhibition of the membrane transporters P-glycoprotein esterases and transferases. The transporters are able to pump back some chemicals to the lumen of the enterocyte^20,25,26^. For the present report it seems pertinent to mention that in mouse it has been described the expression of the enzyme CYP3A11, which correspond to the human CYP3A4 representative^27^, and that even though the metabolic differences, pharmacokinetic studies in mouse have human clinical relevance, because mouse most closely approximates the determination of pharmacokinetic measures in humans than other *in vivo* or *in vitro* model, showing a linearity response greater than 0.94% respect to human studies^28,29^.

On the other hand, ifosfamide (IF) is an anticancer agent used for the treatment of various solid tumors, soft tissue sarcoma, and leukaemia^30^. The compound is an oxazaphosphorine prodrug that requires activation by the hepatic cytochrome P450, in particular a 4-hydroxylation catalyzed by CYP3A4, CYPC9 and CYP2B6 enzymes which yields a cytotoxic nitrogen mustard that can react with the DNA molecule to form crosslinks and lead to cell apoptosis^31^. During its metabolic pathway, the CYP2B6 enzyme may also generate the neurotoxic and nephrotoxic compounds, 2- and 3-dechloroethylifosfamide, respectively^32,33^.

With respect to IF, its nitrogen mustard and conjugates, several transporters related with their uptake and efflux have been described, such as the breast cancer resistance protein and the multidrug resistance associated proteins^31^. Therefore, the involvement of various CYP enzymes in the metabolism of IF, as well as the participation of protein transporters in its absorption, suggest that agents interacting with the antineoplastic may give rise to modifications in its pharmacokinetics, which in turn, may be reflected as alterations in its absorption, distribution, biotransformation and/or excretion^31,34^.

Therefore, considering that knowledge of GFJ and naringin interaction with a variety of drugs may be useful to correct time/dosage of the involved medicament, as well as the absence of information respect to IF, the aim of the present investigation was initially, to validate a method for the quantification of the antineoplastic agent in the plasma of mouse, and subsequently, to determine if mice pretreated with GFJ and/or naringin may modify the IF pharmacokinetics.

## Results

### Validation of the HPLC procedure for the quantification of ifosfamide

For this purpose, the linear regression of the peak-area ratios versus concentrations was fitted in the concentration range of 5–100 μg/mL. A typical equation of the calibration curve on a validation run was as follows:

$$y=0.047x-0.094\,({r}^{2}=0.994)$$

where “*y*” represents the peak-area ratio of the analyte (IF) to the internal standard (CF), and “*x*” represents the plasma concentration of IF. Good linearity was obtained in this concentration range. Total mean (% RSD) recoveries were 101.05% (8.19%), 100.62% (0.55%), and 95.47% (2.94%) for the low, medium, and high QC, respectively. The precision and accuracy values corresponding to low, medium, and high QC are shown in Table 1. These values were within the acceptable range, and the method was thus judged suitable, accurate and precise. The lower limit of quantification of IF was established as 5 μg/mL, with precision of 4.56%, and accuracy of 3.17%.

**Table 1:** HPLC Method for determining ifosfamide (IF) in the plasma of mice. Results on precision and accuracy.

IF concentration	R.S.D (%)		RE (%)
(μg/mL)	Intra-day	Inter-day	
10	11.34	14.92	−4.27
40	8.15	9.89	14.39
90	2.39	7.04	12.83

The relative standard deviation (RSD) shows the deviation of the usual standard deviation when the mean of the data set is compared. $RSD=(\frac{Standard\,deviation}{Mean})(100)$.

The % of the relative error (RE %) is a form to express the accuracy of a determination respect to a central tendency $RE=(\frac{Observed\,concentration-Nominal\,concentration}{Nominal\,Concentration})(100)$ *n* = 3.

The results concerning stability tests of IF are shown in Table 2. These were designed to cover anticipated conditions of handling in the laboratory respect to the IF analysis.

**Table 2:** Stability determination of ifosfamide (IF) under various storage conditions.

Storage conditions	Concentration (μg/mL)		RSD (%)	RE (%)
	Added	Found		
Short-term (24 h, 25 °C)	10	12.31 ± 0.68	5.39	−11.66
	90	100.11 ± 14.27	14.26	0.04
Long-term (30 days, −70 °C)	10	11.17 ± 0.53	4.77	−6.18
	90	108.34 ± 1.64	1.51	−11.15
Freeze and thaw stability (−70 to 25 °C)	10	11.16 ± 0.11	0.99	−1.82
	90	81.34 ± 4.99	6.14	18.78

$$RSD=(\frac{Standart\,deviation}{Average})(100)$$.

$$RE=(\frac{Observed\,concentration-Nominal\,concentration}{Nominal\,Cocentration})(100)$$.

*n* = 3.

### Ifosfamide pharmacokinetic parameters in GFJ and naringin treated mice

**Figure 1:**
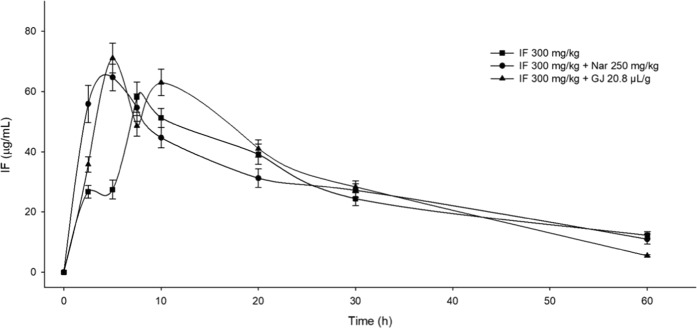
Mean mouse plasmatic concentrations of ifosfamide (IF) respect to time. Mice administered IF, naringin (Nar) plus IF, and grapefruit juice (GFJ) plus IF. Naringin and GFJ were administered 1 h before IF. Each point represents the mean ± SEM of the concentration of IF in the plasm of mice at 2.5, 5, 7.5, 10, 20, 30 and 60 min after its administration.

The developed and validated method was applied to monitor the IF plasmatic levels in the groups also administered naringin or GFJ. By comparing the IF mean concentrations among the different treatments we observed a clear *C*_*max*_ increase, as well as a reduction in the time required to reach such concentration (*T*_*max*_) (Fig. 1). The IF pharmacokinetic analysis of the individual data from the twelve temporal courses was made through a non-compartmental model, therefore, we assumed that values of the seven mice (examined at 2.5, 5, 7.5, 10, 20, 30, and 60 min of exposure) corresponded to a single profile. This approach was appropriate for the statistical analysis of the registered parameters. Table 3 shows a *C*_*max*_ of 58.1 µg/mL for the temporal course of the IF levels; however, when 250 mg/kg of naringin was orally administered 1 h before the anticarcinogen (300 mg/kg), the observed *C*_*max*_ value of IF (64.1 μg/mL) corresponded to a significant increase of 10.4% respect to the value of IF alone. As regards to the effect of GFJ (20.8 µL/g) administered 1 h before IF we also found a statistical difference concerning the observed *C*_*max*_, in this case, the value of 71.3 μg/mL, represent 22.8% of a concentration increase for IF. In connection with *T*_*max*_ results, we observed a significant decrease of this parameter with both, naringin and GFJ. In comparison with the time of 10.8 min for IF, the administration of GFJ plus IF gave rise to 8.7 min for *T*_*max*_, and with respect to the administration of naringin plus IF the result was of 5 min, a time that was even significant with respect to the value of GFJ plus IF.

**Table 3:** Pharmacokinetic parameters obtained in the plasma of mice treated with ifosfamide (IF) alone, and with a previous administration of naringin or grapefruit juice (GFJ).

Measured Parameter	IF only (300 mg/kg)	Naringin + IF (250 mg/kg + 300 mg/kg)	GFJ + IF (20.8 µL/g + 300 mg/kg)
*C*_*max*_ (μg/mL)	58.08 ± 5.01	64.12 ± 4.37^a♣^	71.35 ± 3.57^a♣^
*T*_*max*_ (min)	10.83 ± 1.28	5 ± 0.68^a♦^	8.75 ± 2.05
$AU{C}_{0-}$_∞_ (μg*min/mL)	2110.36 ± 114.98	2245.40 ± 211.9	2052.03 ± 102.33
*t*_*1/2*_ (min)	23.55 ± 1.86	26.77 ± 3.77	16.54 ± 1.43^a♦^

Naringin and GFJ were administered 1 h before IF. n = 3.

Values represent mean ± SEM, this were obtained with the WinNonlin 4.0 software. ^a^Statistically significant difference with respect to the value obtained from treated group with IF (300 mg/kg).

^♣^One way ANOVA, *pos hoc* Student-Newman-Keuls (p ≤ 0.05).

^♦^Kruskal-Wallis, *pos hoc* Student-Newman-Keuls (p ≤ 0.05).

The amount of IF absorbed by mouse after its oral administration (represented by the *AUC*_0−∞_) was equal to 2110.4 μg × min/mL. The values obtained in the treatments of naringin plus IF, and of GFJ plus IF showed no statistical difference with such value, although a slight tendency of naringin to increase the amount of IF, and of GFJ to reduce the *AUC*_0−∞_ of IF was observed. Finally, the half-life (*t*_*1/2*_) of IF was 23.5 min. This value decreased 29.8% when GFJ was administered, however, no change was observed in the case of naringin.

## Discussion

Regarding the study to quantitate the concentration of IF in the plasma of mice, the obtained results suggest that the applied HPLC method was reliable in light of the exactitude and precision of the obtained data, which were within the permitted limits. Besides, the observed data were also reliable because of the confirmation that temperature and storage variations could not be factors that alter the stability of IF. As to GFJ and naringin interaction with IF, it was of interest to note that the *C*_*max*_ values were higher and were reached in shorter times when the two agents (GFJ and naringin) were given before the administration of IF, in comparison with values obtained only with the IF treatment. Interestingly, IF administered alone showed a subtle flattening of the curve followed by a continuous and prolonged elimination phase after reaching the C_*max*_^35,36^. We think that this behavior does not strictly correspond to a biphasic curve but most probably to the multiple peaks phenomenon, which has usually been reported in pharmacokinetic profiles of molecules examined in blood, by action of the particular formulation of the agent, their physicochemical properties or by physiological factors^37^. In light of the fact that the used IF was pure and not a pharmaceutical form, we consider that its curve behavior was mostly related with physiological factors. Such factors usually include the enterohepatic recycling, and they correspond to the effect of biliary secretions on the elimination of the xenobiotic, followed by its intestinal resorption^38^; the biochemical differences among the various regions of the gastrointestinal tract (GT) (regiospecificity in the concentrations of bile and/or proteins transport), which are known to be responsible for the progress of absorption on the GT (secondary absorption site), and consequently with the appearance of the site-specific absorption which are represented by various peaks^39^. Another important element refers to the influence of physiological factors of the GT, such as pH, bile salts, phospholipids, and to the presence of other chemicals. These factors are known to determine the speed of transit in the intestine and have direct repercussion on the absorbance process^40^. Thus, our apparent IF biphasic curve could be originated by one or more of the mentioned factors, and therefore be in line with the monophasic curve previously observed for this chemical^35^.

This type of curves has been observed in a number of pharmacokinetic profiles obtained by administering the compounds in conjunction with GFJ; for example, in the case of rosuvostatin, celiprolol, felodipine, and cyclosporine^41–43^. The explanation for the formation of such curves has relies in the high number of compounds present in GFJ including flavonoids, furanocoumarins, carotenoids and vitamins, chemicals that may interact with the drugs to modify pharmacokinetic parameters^44,45^.

The reported findings indicated that such interaction gave rise to changes in the bioavailability of IF as indicated in the previous section. Regarding *AUC* data, no changes were determined in the cases of GFJ or naringin; however, the *t*_*1/2*_ of IF showed a significant decrease but only with the ingestion of GFJ.

GFJ may increase the plasma concentration of drugs, but mainly those that have a higher phase I metabolism, such as IF^46^. Usually, GFJ does not affect the drug’s half-life, although there are reports that show a decrease of this parameter^47^. Although CYP3A4 is present in liver and intestinal mucosa, GFJ mainly acts in the intestine because their components are degraded before they can reach the hepatic CYP3A4^48^. Consequently, its effect to increase the plasma level of some substances is due to alterations in the normal intestinal absorption process, once the drug is taken up in the mucosa. IF may be metabolized by hepatic CYP3A4 or pumped back into the intestine lumen by P-glycoprotein and OATP^49,50^. However, when GFJ is administered it may inhibits the activity of these transporters causing an accumulation of IF in the blood (increasing its *C*_*max*_). Once in bloodstream, IF reaches the liver, where is metabolized to 4-hydroxyphosphamide by the unchanged liver CYP3A4 (and by the isoforms 2B6, 2A6, 2C8, 2C9, 2C19 in a lesser extent)^51,52^. It is important to note that IF metabolism is characterized by being self-induced, that is, IF auto induces its biotransformation by activating the xenobiotic receptor PXR coded by NR1I2 which mediates its autoinduction by the transcriptional upregulation of CYP3A4^53^. Therefore, the concentration increase of IF by GFJ generates a higher concentration of the anticarcinogen in blood, which may increase the transcriptional induction of CYP3A4, self-increasing its own metabolism over time and, consequently, its elimination rate. Therefore, we suggest that an explanation for the IF *C*_*max*_ increase and its *t*_*1/2*_ decrease its at the intestinal level. At this point, GFJ increases the absorption of non-metabolized IF by inhibiting its intestinal metabolism and pumped back into the intestine lumen. Thus, the high concentration of IF in blood self-induces its own metabolism by CYP3A4 induction and, in consequence, decreases its half-life.

The IF therapeutic potential is well known to include significant DNA damage. On the other hand, naringin has been demonstrated to partially prevent such damage in mouse^54,55^. These authors suggested the pertinence of exploring whether the DNA inhibitory effect of naringin could be related with inhibition of the IF absorption/bioavailability; our present results, however, did not support such possibility. Thus, the antioxidant capacity of naringin remains as the main underlying activity involved in its antigenotoxicity.

At the clinical level, pharmacokinetic changes may have no relevance, as well as moderate or even serious relevance. Respect to the last case, it was observed that a single glass of GFJ (250 mL), or the consumption of fresh fruit segments might reduce presystemic metabolism and increase bioavailability of a number of drugs. When inhibition of P-glycoprotein occurs, it is possible to observe an increase in the drug’s bioavailability related with a decrease of the intestinal or hepatic efflux transport. With respect to the inhibition of organic anion transporting polypeptides, a reduction in the intestinal uptake transport can occur inducing a reduction in the drug’s bioavailability^56,57^. These alterations may affect the patient’s health depending on the involved medication; for example, the interaction of GFJ with ergotamine may cause gangrene or stroke; with nimodipine systemic hypotension, the interaction with digoxin may cause electrical and mechanical disturbances in the heart, and with atorvastatin, lovastatin, or simvastatin the risk of rhabdomyolysis can increase^56,58^.

Various factors can be involved in the patient´s response to the interaction of GFJ or naringin with drugs, such as individual susceptibility and/or the ingested amount of the involved agents. Such conditions may explain the complexity and heterogeneous response of medications respect to the action of GFJ and naringin, a response which goes from various grades of bioavailability modification to the absence of alteration, for example in compounds such as quinine, digoxine, caffeine, and talinolol^58–60^.

## Conclusions

The present study validated for the first time a method for the quantitation of IF in the plasma of mouse, and determined pharmacokinetic changes of such compound in mice pretreated with GFJ or naringin. Regarding the second objective, our results showed modifications in the *C*_*max*_ and *T*_*max*_ of IF. The observed changes, however, do not suggest clinical hazards to patients consuming GFJ in moderate amounts. Nevertheless, in order to reach a definite conclusion it should be advisable to further extent the study using other experimental conditions, including the time and duration of GFJ and naringin administration, the doses tested, and the sampling times.

## Methods

### Chemicals and animals

The compounds IF and cyclophosphamide (CF, internal standard) (USP grade) were obtained from Sanfer Laboratories (Mexico City, Mexico). Naringin (4′,5,5-trihdroxy-flavanone-7-rhamnoglucoside), acetonitrile and triethylamine were purchased from Sigma-Aldrich Chemicals Co. (St. Louis, Mo. USA). Methanol (HPLC grade) and phosphate salts were purchased from J.T. Baker Chemicals (Mexico City, Mexico). GFJ was freshly squeezed fruit, *Citrus paradisi* Macfad. var. Ruby red, cultivated in a pesticide free field in Albeciras, Veracruz, 400 km south-west of Mexico City.

The antineoplastic IF and CF were dissolved in methanol to prepare a standard concentration of 1 μg/μL in each case. Also, phosphate buffer solutions (0.01 N, and 0.025 N) were prepared and adjusted to pH 4.0.

The experimental protocol in mice was approved by the Committee of Ethics and Biosecurity of the National School of Biological Sciences. We used ICR male mice of 25 g obtained from Biotinox (Mexico City, Mexico) which were housed in polypropylene cages at 22 ± 2 °C, 50–60% relative humidity, and under a 12 h light-dark cycle. They were fed with laboratory animal feed (Rodent Lab Chow 5001, Purina) and purified water. Besides, all methods were performed in accordance with the relevant guidelines and regulations.

### Validation of the HPLC procedure for the quantification of ifosfamide

The analytical method was validated according to criteria established by the National norm (NOM-177-SSA1-1998) and International norms (ISO/IEC 25:1990). Mouse IF-free plasma was spiked with IF solutions to obtain a calibration curve at concentrations of 5, 20, 30, 50 and 100 μg/mL. Similarly, quality control samples were prepared at low, medium, and high concentration levels (10, 40, and 90 μg/mL). These were employed to determine absolute recovery, as well as intra- and inter-day precision and accuracy. Precision was expressed by relative standard deviation (RSD), and accuracy as relative error (RE). The intra- and inter-day precision was required to be below 15%, and the accuracy to be within ±15%.

The lower limit of quantification (LLOQ) was determined in five replicates, and corresponded to the lowest concentration of the calibration curve that could be measured with acceptable accuracy and precision. The precision should be equal or less than 20% and accuracy should be within ±20%.

The stability of IF in the plasma of mouse was assessed by analyzing (in triplicate) plasma samples at concentrations of 10 and 90 μg/mL, which were exposed to different conditions (time and temperature). The short-term stability was determined after the exposure of the spiked samples to room temperature for 24 h. The long-term stability was evaluated after storage of the standard spiked plasma samples at −70 °C for 30 days. The freeze/thaw cycle stability was assessed after two complete freeze/thaw cycles (−70 to 25 °C) on consecutive days. The analytes were considered to be stable in plasma when 85–115% of the initial concentrations were found.

Chromatography conditions for the study were as follows: the mobile phase consisted of acetonitrile, potassium phosphate buffer 0.025 M at pH 4.0, and triethylamine (12:88:0.025, v/v) with a final pH of 6.2. The flow-rate was 1.2 mL/min. The analytical column was operated at ambient temperature. The HPLC system consisted of a Beckman System Gold, 128 Solvent Module with ultra-violet detection at 200 nm. We used a 3.5 μm Xterra RP-8 (4.6 × 150 mm) column. Data were collected by the HPLC system and transferred to a Dell Pentium computer.

### Mouse IF pharmacokinetic study

For the study, 7 groups with 12 animals each were intragastrically (ig) administered with 300 mg/kg of IF. Then, we obtained 1 mL of blood by cardiac puncture at 2.5, 5, 7.5, 10, 20, 30, and 60 min post-administration.

Blood samples were immediately centrifuged at 3500 rpm. Aliquots of 250 µL were stored at −70 °C until the quantification of IF plasmatic concentrations was carried out.

### Ifosfamide pharmacokinetic parameters in GFJ and naringin treated mice

Besides in our research, 7 groups with 12 animals each were ig administered with naringin (250 mg/kg) to evaluate its influence on the concentration/time profiles of IF, a chemical that was ig administered 60 min later (300 mg/kg). Seven other groups were ig administered GFJ (20.8 µL/g) and 60 min later ig treated with 300 mg/kg of IF. In both assays, we obtained blood samples at 2.5, 5, 7.5, 10, 20, 30, and 60 min post-administration. With the indicated design we examined the influence of GFJ or naringin on the concentration/time profiles of IF.

For the blood treatment, after thawing, we added 250 μL of the internal standard (CF) to each sample, after which the plasma was deproteinized with methanol 1:1.5 (plasma-methanol), and centrifuged at 3500 rpm for 10 min. Subsequently, we transferred 450 μL of the supernatant to an Eppendorf tube containing 550 μL of phosphate buffer 0.01 N, pH 4.0. The solution was activated with 1 mL of methanol plus 1 mL of phosphate buffer 0.01 N, pH 4.0, and loaded to a Bond Elut-CH cartridge under reduced pressure. The cartridge was washed with 1 mL of acetonitrile plus phosphate buffer 0.01 N, pH 4.0 (10–90%) and it was eluted with 500 μL of acetonitrile plus phosphate buffer 0.025 N, pH 4.0 (40–60%). A volume of 20 μL were injected in the stabilized HPLC system.

### Pharmacokinetic data and statistical assays

The pharmacokinetic parameters of IF and their interaction with naringin or GFJ were calculated by non-compartmental assessment of data using the computer program WinNonlin (V4.0, Pharsight, Mountain View, CA, USA). The maximum plasma concentrations (*C*_*max*_) and their time of occurrence (*T*_*max*_) were both obtained directly from the measured data. The area under the plasma concentration-time curve, from time zero to the time of the last measurable concentration (*AUC*_0-*t*_) was calculated with the linear trapezoidal method. *AUC*_0-∞_ was calculated *AUC*_0-*t*_ + *C*_*t*_/*λ*_*z*_, where *C*_*t*_ is the last measurable concentration and *λ*_*z*_ the constant rate of terminal elimination. The corresponding half-life elimination (*t*_*1/2*_) was then calculated as 0.693/*λ*_*z*_. The results corresponding to *C*_*max*_ were statistically analyzed with the ANOVA and the Student-Newman-Keuls tests. With respect to *AUC*_0-*t*_, *T*_*max*_, and *t*_*1/2*_ the analysis was made with the Kruskal-Wallis test.
